# Eradicating Malaria by Way of the Human Host

**Published:** 1916-03

**Authors:** 


**Eradicating Malaria by Way of the Human Host.** Japan decided to combat malaria in Formosa, not by filling up ponds, etc., and thus diminishing the anopheles, but by rounding up the human inhabitants and exterminating the protozoa in them. 996,621 persons were examined and 11,896 carriers were found and treated with quinine. In two districts the malaria mortality has been reduced to zero, from rates of 15:1000 and 5:1000 respectively. In another, the reduction has been from 11.60 to 3.39:1000—all in two years. This is an exemplification of the "Man at a Time" doctrine. Essentially the same results have been obtained, according to our Associate Editor, Dr. Pel, in Holland, not from any formal campaign but simply in the course of ordinary intelligent therapeutics. There are many localities in which the filling up of breeding places or the use of kerosene or crude oil, etc., is of prohibitive magnitude or undesirable for economic reasons. The results noted seem to settle the question as to whether, in a practical sense, the primary host is man or the anopheles, and eradication by way of the human host is a simpler and more directly humanitarian method.—Practical Medicine, Calcutta.

